# Pulmonary Valve Endocarditis With Mycotic Pulmonary Aneurysm Revealing a Previously Undiagnosed Perimembranous Ventricular Septal Defect in an Adult

**DOI:** 10.7759/cureus.92008

**Published:** 2025-09-10

**Authors:** Mehdi Berrajaa, Hala Jaouhari, Amine El Houari, Wassim Beladel, Khalil Abderrahmane Elbaz, Mohamed El Minaoui

**Affiliations:** 1 Cardiology Department, Souss-Massa University Hospital, Faculty of Medicine and Pharmacy, University Ibn Zohr, Agadir, MAR

**Keywords:** adult congenital heart disease, endocarditis, perimembranous ventricular septal defect, pulmonary artery aneurysm, pulmonary valve

## Abstract

Infective endocarditis (IE) is a severe infection affecting the endocardial surfaces, including the heart valves and chordae tendineae. Ventricular septal defect (VSD), a common congenital heart defect, is associated with an elevated risk of developing IE. Pulmonary valve infective endocarditis (PVIE) is rare, especially in patients without typical risk factors, and is associated with high morbidity and mortality if not diagnosed and treated promptly. We present here a rare case of PVIE complicated by a pulmonary artery aneurysm, which led to the incidental discovery of a perimembranous VSD in a 33-year-old man. Transthoracic echocardiography (TTE) and transesophageal echocardiography (TEE) revealed the VSD with pulmonary valve (PV) vegetations, while CT imaging identified a mycotic aneurysm and splenic infarction. Blood cultures confirmed *Streptococcus pharyngis* as the causative pathogen. The patient received intravenous antibiotics, resulting in significant clinical improvement, with the resolution of fever and the normalization of laboratory parameters. This case underscores the importance of the early diagnosis and management of PVIE, as well as the need to consider underlying congenital heart defects.

## Introduction

Infective endocarditis (IE) is an infection of the endocardium, including the heart valves and chordae tendineae [1]. Ventricular septal defect (VSD) is a common congenital cardiac anomaly that increases the risk of IE [2]. Although the aortic valve is most frequently affected, right-sided IE is increasingly recognized due to its rising incidence [3], particularly in patients with congenital heart defects such as VSD [4]. In VSD with left-to-right shunting, IE can cause septic emboli to enter the pulmonary circulation, potentially leading to mycotic pulmonary artery aneurysms. Although uncommon, these aneurysms represent a serious complication due to their risk of rupture and massive hemorrhage [3]. Management typically involves prolonged intravenous antibiotic therapy, with surgical intervention considered in cases of persistent infection or structural damage [5]. Here, we report a case of pulmonary valve infective endocarditis (PVIE) complicated by a mycotic pulmonary artery aneurysm, which led to the incidental diagnosis of a perimembranous VSD in an adult.

## Case presentation

**Figure 1:**
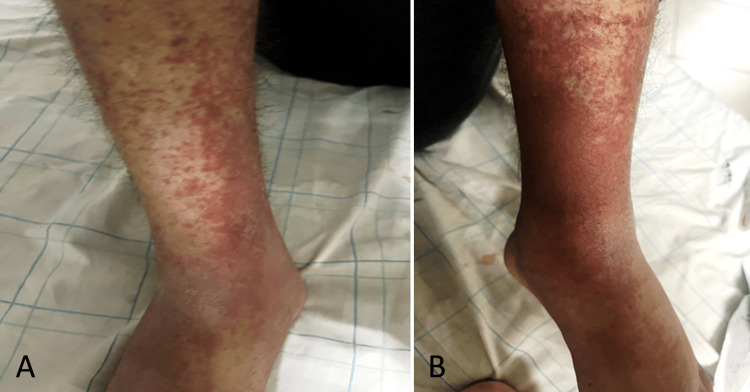
Petechial purpura of vascular origin on both lower limbs (A and B).

We present herein the case of a 33-year-old man with a history of type 2 diabetes, with complete vision loss eight years ago due to diabetic retinopathy. The patient had no significant cardiac history. He presented with asthenia, low fever, and arthralgia of the large joints, evolving over the past 20 days. Five days prior to admission, his symptoms worsened with the onset of shortness of breath (New York Heart Association {NYHA} stage II dyspnea), persistent palpitations, and a purpuric rash on both lower limbs, which prompted him to seek medical care. Physical examination revealed tachycardia at 145 beats per minute (bpm), with normal blood pressure of 135/74 mmHg. On auscultation, a right-sided holosystolic murmur was noted, along with basilar crepitations. Dermatological examination showed a rash on both lower limbs (Figure 1).

The rest of the physical examination, including abdominal and neurological assessment, was unremarkable. The ECG showed sinus tachycardia at 149 bpm, without any other significant abnormalities. Given the patient's clinical presentation with infectious symptoms (fever, rash, arthralgia, and asthenia), tachycardia, and abnormal heart sounds, the diagnosis of infective endocarditis was strongly suspected, warranting an echocardiographic examination.

**Figure 2:**
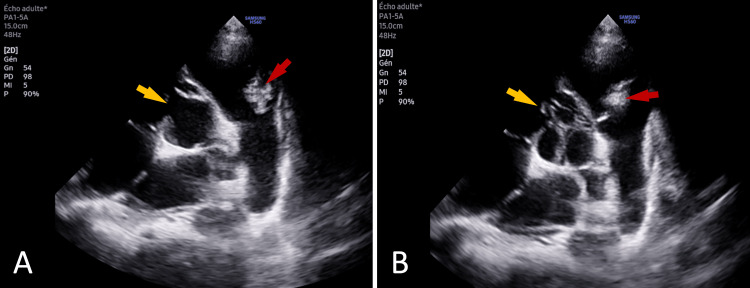
Transthoracic echocardiographic view in the parasternal short axis showing a perimembranous ventricular septal defect (yellow arrows) with a large vegetation on the pulmonary valve (red arrows), during systole (A) and during diastole (B).

Transthoracic echocardiography (TTE) revealed a perimembranous ventricular septal defect (VSD) with a left-to-right shunt (a gradient of 52 mmHg), as well as a pulmonary valve (PV) bearing multiple vegetations, the largest measuring 11.8 × 7 mm, leading to severe pulmonary regurgitation (Figure 2A, 2B). The echocardiogram also demonstrated a dilated left atrium and right ventricle, along with a small pericardial effusion.

**Figure 3:**
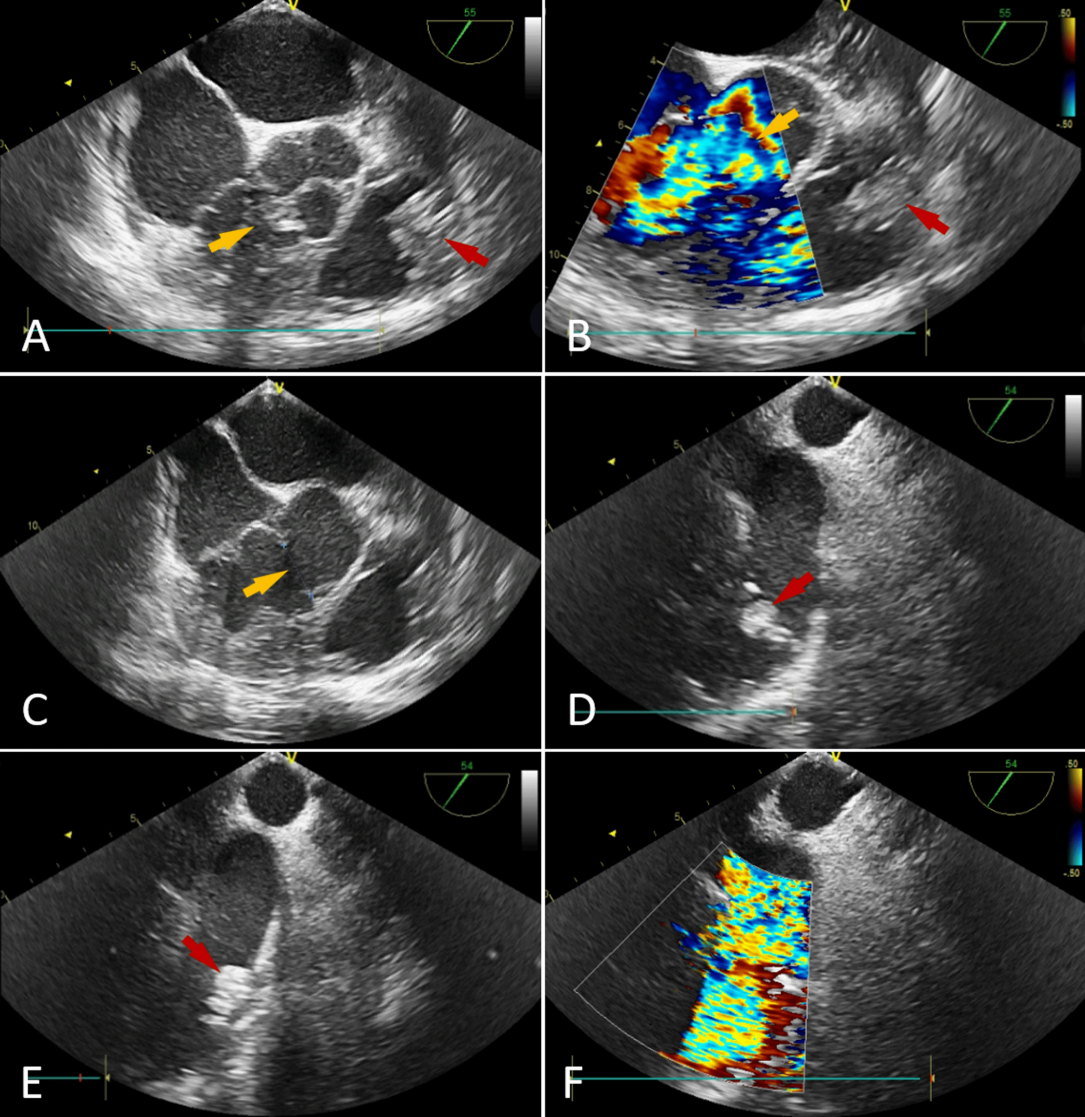
Transesophageal echocardiographic view. Mid-esophageal aortic valve short axis showing a perimembranous ventricular septal defect (yellow arrow) with a large vegetation on the pulmonary valve (red arrow) (A). Mid-esophageal aortic valve short axis showing a perimembranous ventricular septal defect (yellow arrow) with left-to-right shunting on color Doppler, along with a large vegetation on the pulmonary valve (red arrow) (B). Mid-esophageal aortic valve short axis showing a perimembranous ventricular septal defect measuring 19 mm (yellow arrow) (C). Upper-esophageal aortic arch short axis showing a large vegetation (red arrows) on the pulmonary valve (D and E). Upper-esophageal aortic arch short axis showing severe pulmonary regurgitation (F).

**Figure 4:**
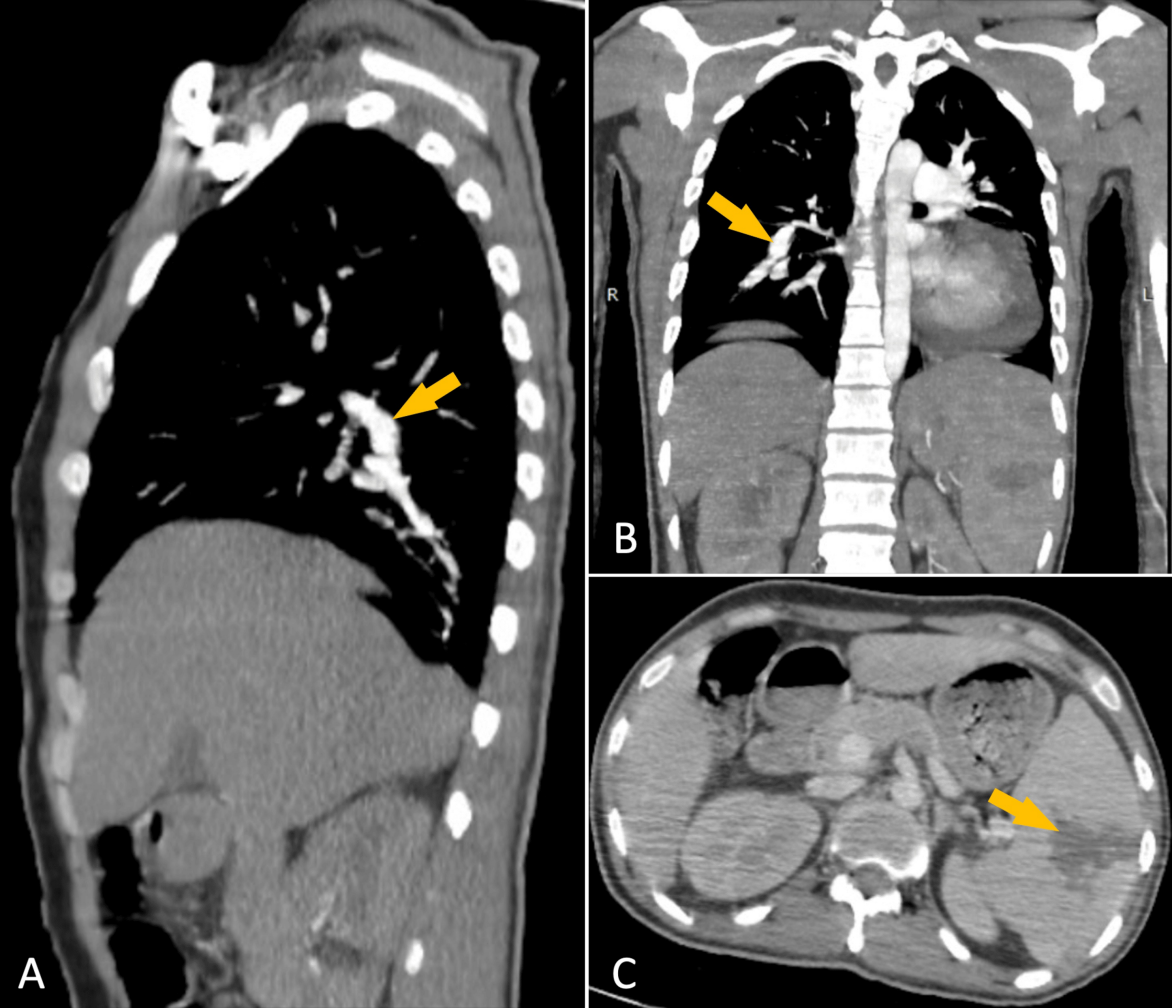
Thoracic and abdominal CT scan. Sagittal (A) and coronal (B) views reveal a mycotic aneurysm of the superior segmental artery of the right upper lobe (arrows). A transverse section (C) demonstrates a splenic infarction.

Transesophageal echocardiography (TEE) further assessed the pulmonary valve dysfunction, confirming the presence of vegetations (Figure 3A, 3B, 3D, 3E) and severe regurgitation, as well as revealing accelerated transvalvular flow (Figure 3B, 3F). Functional stenosis was evidenced by a maximum velocity of 4.9 m/s and a peak gradient of 98 mmHg, associated with a 19 mm ventricular septal defect (VSD) (Figure 3A, 3B, 3C). The left ventricle had normal dimensions and function, without hypertrophy, and the aortic and mitral valves appeared unremarkable. Thoracic and abdominal CT scans revealed a mycotic aneurysm of the superior segmental artery of the right upper lobe (Figure 4A, 4B) and a splenic infarction (Figure 4C).

Laboratory investigations showed low hemoglobin levels at 7.5 g/dL, a high white blood cell count of 25,580/µL, elevated neutrophils at 21,794/µL, and increased platelets at 745,000/µL. C-reactive protein (CRP) was markedly elevated at 139.9 mg/L, supporting the presence of an underlying infectious process. Renal dysfunction was noted with urea at 1.04 g/L, creatinine at 35.85 mg/L, and a reduced glomerular filtration rate of 27 mL/minute/1.73 m² (Table 1). Blood cultures were positive for *Streptococcus constellatus *subsp. *pharyngis*, which was sensitive to vancomycin and gentamicin. The diagnosis of pulmonary valve infective endocarditis (PVIE) was confirmed according to the modified Duke's criteria.

**Table 1:** Laboratory results of our patient. LDL, low-density lipoprotein; HDL, high-density lipoprotein

Parameter	Result	Normal Range
Complete Blood Count		
Hemoglobin (Hb)	7.5 g/dL	13-17 g/dL (Male)
White Blood Cell Count	25,580/µL	4,000-10,000/µL
Neutrophils	21,794/µL	2,000-7,500/µL
Lymphocytes	2,200/µL	1,000-4,000/µL
Platelets	745,000/µL	150,000-400,000/µL
Inflammatory Markers		
C-reactive Protein (CRP)	139.9 mg/L	<5 mg/L
Renal Function		
Urea	1.04 g/L	0.15-0.45 g/L
Creatinine	35.85 mg/L	6-12 mg/L
Glomerular Filtration Rate	27 mL/minute/1.73 m²	>90 mL/minute/1.73 m²
Liver Function Tests		
Aspartate Aminotransferase	26 IU/L	10-40 IU/L
Alanine Aminotransferase	31 IU/L	10-45 IU/L
Blood Electrolytes		
Potassium	4.74 mmol/L	3.5-5.0 mmol/L
Sodium	135 mmol/L	135-145 mmol/L
Chloride	98 mmol/L	95-105 mmol/L
Calcium	83 mg/L	85-105 mg/L
Magnesium	24 mg/L	18-24 mg/L
Coagulation Profile		
Prothrombin Time	70%	70%-100%
Activated Partial Thromboplastin Time (aPTT)	1 (Ratio)	0.8-1.2 (Ratio)
Glycated Hemoglobin (HbA1c)	6.7%	<5.7%
Lipid Profile		
LDL Cholesterol	0.11 g/L	<1.3 g/L
HDL Cholesterol	0.12 g/L	>0.4 g/L
Total Cholesterol	0.67 g/L	<2.0 g/L
Triglycerides	1.76 g/L	<1.5 g/L
Immunodeficiency Workup		
HIV and Syphilis Serology	Negative	Negative
Blood Cultures (Hemocultures)	3 positive cultures	Negative
Identified Organism	Streptococcus pharyngis	-
Rheumatoid Factor	Positive	Negative

The patient received a six-week course of intravenous antibiotics (ceftriaxone and gentamicin, owing to the initial unavailability of vancomycin, with no antifungal therapy administered) and showed a favorable clinical response with the resolution of fever and the normalization of laboratory parameters. However, follow-up imaging revealed persistent vegetations and severe regurgitation.

In the context of pulmonary valve endocarditis complicated by a pulmonary artery mycotic aneurysm, the discovery of a perimembranous VSD, and severe pulmonary regurgitation, surgery was proposed after discussion with the family; however, the patient declined. He remains clinically stable, with normal inflammatory laboratory parameters, but continues to refuse surgery at this time.

## Discussion

Infective endocarditis is an infectious process involving the endocardium; multiple structures could be affected, including the valves and chordae tendineae, and even foreign materials such as prosthetic valves and implanted devices [5]. Accounting for up to 40% of congenital heart defects, a ventricular septal defect is one of the most frequent cardiac congenital malformations [2]. Perimembranous defects result from the incomplete closure of the membranous septum and adjacent muscular components [6,7]. The modified Duke's criteria for IE diagnosis are based on multiple criteria, including clinical, echocardiographic, and infective investigations [8]. Findings such as anemia, leukocytosis, and elevated inflammatory markers should prompt the consideration of infective endocarditis [9].

Right-sided IE is becoming increasingly more recognized, due to the increased morbidity and mortality [4]. PVIE is a rare condition, accounting for up to 2% of all cases of IE [4]. The infection may be limited to the pulmonary valve or spread to adjacent cardiac structures [10]. A study done by Isaza et al. included 2,124 cases of IE and showed that PVIE represented only 1.1% of cases, with half of the cases occurring in patients with prosthetic valves. Clinical manifestations usually include nonspecific symptoms linked to the infective process, such as nausea, vomiting, and fever [4].

As recommended by the American Heart Association (AHA) guidelines, both TTE and TEE are important during the initial evaluation and subsequent follow-up of IE [10]. TTE has a higher sensitivity and specificity, respectively, 87%-100% and 91%-100%, compared to TTE [11]. It usually reveals the presence of vegetation, which represents the hallmark of this affection. However, it is difficult to visualize the pulmonary valve, and it is possible to miss endocarditis involving this valve [9]. Cardiac magnetic resonance imaging (MRI) and CT angiography are valuable tools for detecting associated complications such as pseudoaneurysms, valve perforations, or abscesses. Additionally, positron emission tomography (PET) can aid in the diagnosis of right-sided endocarditis [12].

Biological investigation usually shows an inflammatory reaction with elevated white blood cells and CRP. The most common pathogens implicated in infective endocarditis are *Staphylococcus aureus* and *Streptococcus* species [13]. In our case, *Streptococcus constellatus* subsp. *pharyngis* was isolated from blood cultures, an uncommon organism belonging to the *Streptococcus anginosus* group. This group is more frequently encountered in immunocompromised individuals, and in our patient, diabetes may have contributed as a predisposing factor.

IE is more often linked to cardiac devices and valvular prostheses [14]. The aortic valve is the most commonly affected by IE, while the pulmonary valve is the least commonly affected [4]; this instance could be explained by lower pressures and oxygen content of blood across the right-sided valves [4] and also due to lesser abnormalities in the PV [10]. Multiple factors have been described as predisposing to IE, including rheumatic heart disease, degenerative valve disease, diabetes, intravenous drug use, and congenital heart disease [4]. A study done by Sharma and Malavia showed that the dominant etiology underlying cardiac malformations included ventricular septal defects, tetralogy of Fallot, and pulmonary valve stenosis [8]. In our case, the undiagnosed VSD and also the immunocompromised state due to diabetes were likely the predisposing factors.

Infective endocarditis is linked to high mortality and morbidity, with an overall 20% mortality in the first month of evolution [14]. However, PVIE is considered a less severe form of IE, which can be medically managed with better outcomes compared to left-sided IE [4]. Mycotic pulmonary aneurysms are dilatations of the pulmonary artery resulting from the infection of the vessel wall [13]. They have been described after right heart endocarditis and are attributed to septicemia or due to septic emboli, where vegetation fragments detach and undergo embolization through the bloodstream [13]. Mycotic pulmonary artery aneurysms are a rare complication associated with ventricular septal defects. In our case, the patient showed multiple locations of septic emboli, including the lungs and spleen.

The AHA guidelines recommend prioritizing a conservative management strategy for the right-sided IE, if possible [10]. Management is primarily based on antibiotic therapy tailored to blood culture results, aiming for rapid infection control and to prevent its spread. Early surgery should be considered in the presence of vegetations with high mobility, recurrent septic pulmonary emboli, or the failure of intensive medical therapy [8]. Managing complications such as pulmonary aneurysms could be challenging as they are unstable and can easily rupture. It is best achieved by surgical procedures such as aneurysmectomy through the complete removal or obliteration of the aneurysm, aneurysmorrhaphy, banding, or lobectomy [8]. In our patient, with the non-resolution of vegetations, even with intensive medical therapy, and also given the underlying etiology of a septal defect, surgical management was highly recommended to obtain a full resolution of the condition.

IE remains a serious and life-threatening condition that is associated with high morbidity and mortality rates if it is not diagnosed and treated promptly [13].

## Conclusions

Pulmonary valve infective endocarditis is a serious condition; although uncommon, it can result in severe complications such as mycotic pulmonary aneurysms. Early diagnosis through clinical assessment and advanced imaging is crucial for effective management. This case highlights a rare and complex association of pulmonary valve endocarditis, mycotic pulmonary artery aneurysm, and a previously undiagnosed perimembranous VSD incidentally identified. It underscores the critical role of comprehensive imaging, especially echocardiography and CT angiography, in evaluating right-sided endocarditis. In patients lacking risk factors, it is important to maintain a high index of suspicion to uncover underlying congenital heart disease.
